# Efficacy and safety of intravenous iron repletion in patients with heart failure: a systematic review and meta-analysis

**DOI:** 10.1007/s00392-023-02207-2

**Published:** 2023-04-19

**Authors:** Davor Vukadinović, Amr Abdin, Insa Emrich, P. Christian Schulze, Stephan von Haehling, Michael Böhm

**Affiliations:** 1 Klinik Für Innere Medizin III, Kardiologie, Angiologie Und Internistische Intensivmedizin, Universitätsklinikum Des Saarlandes, Universität Des Saarlandes, Kirrberger Strasse, 66421 Homburg/Saar, Germany; 2 Department of Internal Medicine I, Division of Cardiology, Angiology and Intensive Medical Care, Friedrich-Schiller-University, University Hospital Jena, Jena, Germany; 3 Department of Cardiology and Pneumology, Georg-August-University, University Medical Centre, Göttingen, Germany; 4 German Center for Cardiovascular Research (DZHK), Partner Site, Göttingen, Germany

**Keywords:** Iron repletion, Heart failure, Ferric carboxymaltose, Ferric derisomaltose

## Abstract

**Introduction:**

AFFIRM-AHF and IRONMAN demonstrated lower rates of the combined endpoint recurrent heart failure (HF) hospitalizations and cardiovascular death (CVD) using intravenous (IV) ferric carboxymaltose (FCM) and ferric derisomaltose (FDI), respectively in patients with HF and iron deficiency (ID) utilizing prespecified COVID-19 analyses.

**Material and methods:**

We meta-analyzed efficacy, between trial heterogeneity and data robustness for the primary endpoint and CVD in AFFIRM-AHF and IRONMAN. As sensitivity analysis, we analyzed data from all eligible exploratory trials investigating FCM/FDI in HF.

**Results:**

FCM/FDI reduced the primary endpoint (RR = 0.81, 95% CI 0.69–0.95, *p* = 0.01, *I*^2^ = 0%), with the number needed to treat (NNT) being 7. Power was 73% and findings were robust with fragility index (FI) of 94 and fragility quotient (FQ) of 0.041. Effects of FCM/FDI were neutral concerning CVD (OR = 0.88, 95% CI 0.71–1.09, *p* = 0.24, *I*^2^ = 0%). Power was 21% while findings were fragile with reverse FI of 14 and reversed FQ of 0.006. The sensitivity analysis from all eligible trials (*n* = 3258) confirmed positive effects of FCM/FDI on the primary endpoint (RR = 0.77, 95% CI 0.66–0.90, *p* = 0.0008, *I*^2^ = 0%), with NNT being 6. Power was 91% while findings were robust (FI of 147 and FQ of 0.045). Effect on CVD was neutral (RR = 0.87, 95% CI 0.71–1.07, *p* = 0.18, *I*^2^ = 0%). Power was 10% while findings were fragile (reverse FI of 7 and reverse FQ of 0.002). Rate of infections (OR = 0.85, 95% CI 0.71–1.02, *p* = 0.09, *I*^2^ = 0%), vascular disorder (OR = 0.84, 95% CI 0.57–1.25, *p* = 0.34, *I*^2^ = 0%) and general or injection-site related disorders (OR = 1.39, 95% CI 0.88–1.29, *p* = 0.16, *I*^2^ = 30%) were comparable between groups. There was no relevant heterogeneity (*I*^2^ > 50%) between the trials for any of the analyzed outcomes.

**Conclusions:**

Use of FCM/FDI is safe and reduces the composite of recurrent HF hospitalizations and CVD, while effects on CVD alone are based on available level of data indeterminate. Findings concerning composite outcomes exhibit a high level of robustness without heterogeneity between trials with FCM and FDI.

**Graphical Abstract:**

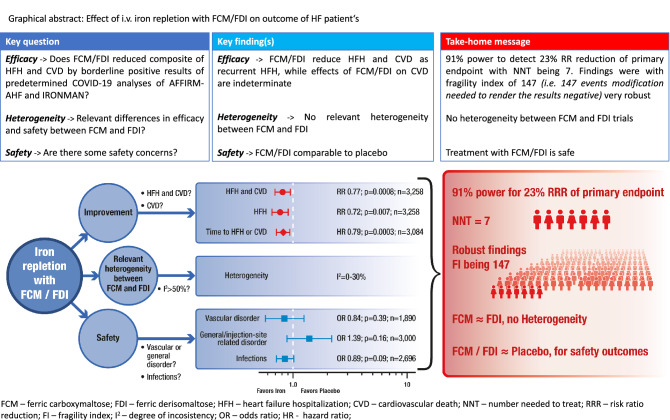

## Introduction

Iron deficiency (ID) frequently occurs in patients with heart failure (HF) being prevalent in up to 50% of patients. [1, 2] Beyond its connection with frailty, impaired quality of life, exercise and functional capacity [3, 4] ID associates with recurrent HF hospitalizations, CVD and all-cause mortality [5]. These associations exist independently of the presence of anaemia [6–8].

Results from randomized trials not powered for clinical end-points have shown that iron supplementation with FCM is safe and improves quality of life, [9, 10] symptoms [9] and exercise capacity[9] in ID, being of the very few HF drugs that have shown benefit in this regard in a recent systematic review [11]. The CONFIRM-HF trial [9] with FCM showed a reduced risk of hospitalizations for worsening HF. This trial was underpowered to determine this clinical outcome. In the powered AFFIRM-AHF trial [12], FCM lowered the rate of composite of total HF hospitalizations and CVD, of note, in a pre-specified COVID-19 sensitivity analysis. Similarly, in IRONMAN [13] treatment with IV ferric derisomaltose (FDI) lowered the same primary endpoint, also in a COVID-19 sensitivity analysis.

In the crude analyses of AFFIRM-AHF and IRONMAN trial, there were only trends for benefit regarding all-cause death or CVD. AFFIRM-AHF and IRONMAN were powered, randomized trials with similar design, identical primary endpoints and both hampered by COVID-19 pandemic [14]. Therefore, we meta-analyzed data from both trials (from crude analyses and those reported in the pre-specified COVID-19 sensitivity analyses) as also data from all eligible trials with similar design to explore whether primary endpoint and CVD is heterogenous between the trials, data were robust and whether the trials had sufficient power to detect the observed effect size. This would strengthen the level of evidence that i.v. iron supplementation improves outcome in HF patients and reassure physicians to screen and treat HF patients for ID.

## Material and methods

### Search and selection strategy

We conducted a meta-analysis of the published randomized controlled trials (RCTs) in accordance with the Preferred Reporting Items for Systematic Review and Meta-Analyses (PRISMA) statement [15]. The protocol for this analysis has been submitted to PROSPERO for registration (CRD 400,041). The search has been performed in MEDLINE and Embase via OVID^@^ using the following keywords and Medical Subject Headings (MeSH) terms: [Heart failure AND iron deficiency OR iron repletion OR intravenous iron OR ferric carboxymaltose OR iron derisomaltose OR iron supplementation OR iron therapy AND randomized controlled trial]. The search was restricted to full-text articles published in English between 2000 and 2022. Furthermore, we screened the reference list of the current guidelines for HF of the European Society of Cardiology [7]. Randomized, placebo-controlled cardiovascular clinical trials that investigated effects of IV iron repletion using ferric carboxymaltose or derisomaltose in patients with HF and ID were considered eligible for inclusion. There was no limit regarding number of patients or duration of follow-up in potentially acceptable studies. Finally, data from non-randomized trials, registries or trials that used oral iron substitution were not considered eligible for inclusion. A reference manager software (Zotero) was used for duplicates removal and data management. Two reviewers (DV and AA) reviewed the full texts and used the same template to extract data relevant to the analysis.

### Data extraction and analysis

Two authors (DV, AA) extracted all data of interest according to a previously established pattern and evaluated the risk of bias at the study level according to the Cochrane risk-of-bias tool (RoB 2.0) [16]. A publication bias would have been assessed using Funnel plot, but only in cases where outcomes of interest were reported in at least ten studies. We extracted the following data: i) baseline characteristics (study design, primary outcome, duration of follow-up, sample size, comparator, regimen of iron substitution, included population); ii) number of events and crude point estimates like rate ratios (RRs) or hazard ratios (HRs) with its associated confidence intervals for outcome of interest. We explored following outcomes of interest: (i) recurrent outcomes (total HF hospitalizations and CVD, total HF hospitalizations), time-to-event outcomes (time to first HF hospitalization or CVD, time to cardiovascular death), (iii) dichotomous outcomes concerning efficacy (all-cause mortality and CVD) but also concerning safety (infections, general or injection-site related disorder, vascular disorder).

We performed a study-level, pairwise meta-analysis based on the intention-to-treat analysis of the summary data exploring the risk of above identified outcomes of interest between the groups (with iron supplementation vs. usual care). In our main analysis, we pooled the data from the AFFIRM-AHF and IRONMAN trials. We compared categorical data from populations included in these trials in terms of age, symptoms, medical treatment and others by applying Pearson’s Chi-squared Test. For the composite endpoint consisting of recurrent HF hospitalizations and CVD we computed corresponding number needed to treat for benefit (NNT) or needed to treat for harm (NNH) as appropriate. We performed the following sensitivity analyses: i) running the analysis by pooling the data from COVID-19 prespecified analyses from AFFIRM-AHF and IRONMAN, (ii) running the analysis by pooling the data from all eligible trials.

For recurrent outcomes, we pooled RRs and for time-to-event outcomes we pooled HRs, which were reported in the original publications. For this purpose inverse variance statistical method was applied. In case RR was not reported, we used number of events to calculate it. For dichotomous outcomes we determined odds ratios (ORs) by applying the Mantel–Haenszel method. The data from each trial were pooled using random-effects (DerSimonian-Laird) model. Heterogeneity between the trials was assessed using Cochran’s Q test and I^2^ statistic. Relevant statistical heterogeneity was considered in case Cochran’s Q-test *p* < 0.05 and *I*^2^ greater than 50%. Study-specific and summary effect estimate with corresponding 95% confidence intervals (CIs) and p-value were visualized using Forest plots.

We explored the robustness of the meta-analysis findings for the composite endpoint (HF hospitalization and CVD) and CVD by determination of the fragility index (FI) for significant outcomes and reversed fragility index (RFI) for non-significant outcomes. We determined FI and RFI by applying the calculator available online http://clinicalepidemio.fr/fragility_ma/. [17] FI indicates the number of specific events-status modification (events added or subtracted in the treatment or placebo group) needed to turn the statistically significant to statistically non-significant results. RFI indicate the number of specific events-status modification needed to turn the statistically non-significant to significant results. Furthermore, we calculated the fragility quotient (FQ) and reversed FQ (RFQ) by dividing FI or RFI respectively with the sample size to account for different sample sizes. FQ represent the proportion of events, which need to be moved to change the significance of results. For example, meta-analysis A had FI of 2 and sample size of 500 participants while meta-analysis B had FI of 2 and sample size of 1000. Albeit FI is the same in both analyses, FQ can reveal us which analysis is relatively more fragile. Analysis A had FQ of 0.004 indicating that 4 events per 1000 patients will be needed to change the results significance; while analysis B has FQ of 0.002 indicating that 2 events per 1000 patients will be needed to change the results significance. Accordingly, FQ suggest us that results of trial B are more fragile.

Lower FI or RFI suggests less statistical robustness although there are no standardized cut off values that defines robustness or fragility. For the purpose of this analysis, FI and RFI < 20 was considered as fragile, FI and RFI 20—40 was considered as moderately robust and FI or RFI ≥ 40 as robust findings.

We calculated the power of our meta-analysis for observed risk ratio reduction (RRR) for composite of recurrent HF hospitalizations and CVD and odds/hazard ratio reduction (ORR/HRR) for CVD in the main and both sensitivity analyses [18].

All statistical analyses were performed using RevMan Version 5.4 and GraphPad Prism Version 6. All *P* values were two-sided, with *P* less than 0.05 considered as significant. For determination of the power of the analysis we used statistic program R Version 4.2.2.

## Results

**Fig. 1:**
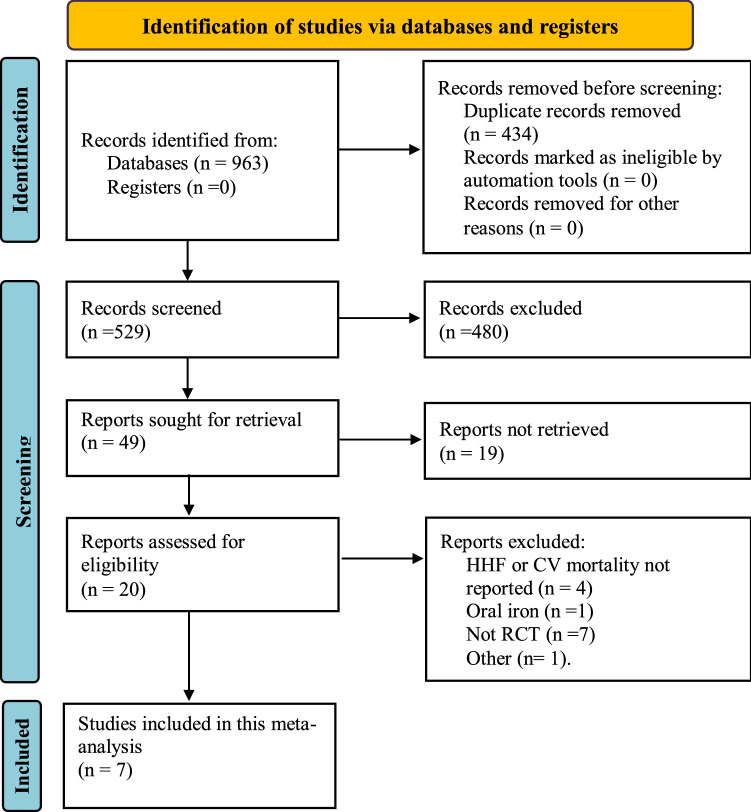
PRISMA flowchart for the studies included and reasons for studies excluded from the systematic review. *CV* cardiovascular, *HHF* heart failure hospitalization, *RCT* randomized control trial

The selection strategy of the statistical analysis is shown in Fig. 1. Finally, 7 studies met the predefined inclusion criteria and were considered eligible for meta-analysis. In our main analysis we explored data from 2,245 participants from the AFFIRM-AHF [12] and IRONMAN [13] trials. For the purpose of sensitivity analysis we explored data from 2171 participants from the COVID-19 sensitivity analyses reported in the AFFIRM-AHF and IRONMAN trials and from 3258 participants by including additional data from the FAIR-HF[9], CONFIRM-HF [10], EFFECT-HF [19] trials and from two small studies (FER-CARS-01 and EFFICACY-HF), whose data were extracted from an article published by Anker et al. [20] as study level data are not published yet. Recurrent and time-to-event outcomes were reported as rates per 100 patient-years in AFFIRM-AHF, IRONMAN and analysis by Anker et al. [20]. Trials FAIR-HF, CONFIRM-HF, EFFECT-HF, AFFIRM-AHF and IRONMAN were regarded as high quality trials (Figure S1, Supplement), for two not published trials (FER-CARS-01 and EFFICACY-HF) evaluations could not be performed for lack of information. Baseline characteristics of the included trials are visualized in Table 1. Evaluation of publication bias was not performed due to futility (less than 10 studies included).

**Table 1:** Baseline characteristics of the included trials

	FER-CARS-01*	FAIR-HF	EFFICACY-HF*	CONFIRM-HF	EFFECT-HF	AFFIRM-AHF	IRONMAN
Patient population	Ambulatory, optimally treated systolic CHF with ID, NYHA class II/III	Ambulatory, optimally treated, systolic CHF with ID, NYHA class II/III, EF < 40% (NYHA II) or EF < 45% (NYHA III)	Ambulatory, optimally treated, systolic CHF with ID, NYHA class II/III	Ambulatory, optimally treated, systolic CHF with ID, NYHA class II/III, LV EF ≤ 45%	Ambulatory, optimally treated CHF with ID, NYHA class II/III, LV EF ≤ 45%	Hospitalised patients for acute HF treated with i.v. furosemide, LV EF < 50%	Hospitalised patients (current or within last 6 months) due to HF or ambulatory patients with HF and raised natriuretic peptides and ID, LV EF ≤ 45%
Randomization	2:1	2:1	1:1	1:1	1:1	1:1	1:1
Sample size (Intervention/placebo)	30/15	304/155	20/14	150/151	88/86	558/550	569/568
Study duration (weeks)	12	24	24	52	24	52	140
Comparator	FCM vs placebo	FCM vs placebo	FCM vs placebo	FCM vs placebo	FCM vs placebo	FCM vs placebo	FDI vs usual care
Calculation of iron repletion dose	Ganzoni formula using the mean of two baseline Hb values	Ganzoni formula using the mean of two baseline Hb values	Ganzoni formula using the mean of two baseline Hb values	Based on baseline Hb and body-weight values	Based on baseline Hb and body-weight values	Based on baseline Hb and body-weight values	Based on baseline Hb and body-weight values
Iron Supplementation (correction and maintenance phase)	– Weekly 200/100 mg FCM i.v. injections for 3-9 weeks - 4-weekly 200 mg i.v. FCM up to 24 weeks	– 200/100 mg weekly for 4 weeks, –every 4 weeks 200 mg if ferritin not > 800 μg/l or ferritin 500–800 μg/l and TSAT > 50% or Hb > 16 g/dL	- Weekly 200/100 mg i.v. injections for 3-9 weeks - 4-weekly 200 mg i.v. up to 24 weeks	- 500/1000 mg at baseline and at week 6 - at weeks 12, 24, 36, additional 500 mg if ferritin < 300 μg/l	-500/1000 mg at baseline - 500/1000 mg at week 6 and week 12 if ferritin < 300 μg/l	- 500/1000 mg at baseline - 500/1000 mg at week 6 and 500 mg at week 12 and 24 if Hb < 15 g/dL	- 1000/2000 mg at baseline - 1000–2000 mg every 4 weeks in case ferritin < 400 μg/L or TSAT < 25%
Primary endpoint	PGA at week 12 and NYHA class from baseline to week 12	PGA at week 24 and NYHA class from baseline to week 24	Change in 6MWT and NYHA class from baselint to week 24	Change in 6MWT from baseline to week 24	Change in peak oxygen consumption (VO2)	Composite of recurrent events of HF hospitalization and CV death	Composite of recurrent events of HF hospitalization and CV death
Link to the public trial registry	NA	NCT00520780	NCT00821717	NCT01453608	NCT01394562	NCT02937454	NCT02642562

^*^study level data not published yet, data extracted from the individual patient-level meta-analysis published by Anker et al. *EHJ 2018*, *CHF *chronic heart failure, *NYHA* New York Heart Association, *ID* Iron deficiency, FCM-ferric carboxymaltose, *FDI* ferric derisomaltose, *LV EF *left ventricle ejection fraction, *NA *not applicable, *6MWT* 6 min walking test

Patient’s characteristic and their medical treatment included in the usual care/placebo group and treatment group differed in the IRONMAN and AFFIRM-AHF trials (Table 2 and S1, Supplement). Usual care population from IRONMAN compared to AFFIRM-AHF study included more men (72% vs 55%, *p* < 0.0001) and fewer women (28% vs 45%, *p* < 0.0001), more patients with New York Heart Association (NYHA) class II (56% vs 42%, *p* < 0.0001) and fewer with NYHA class III (42% vs 50%, *p* = 0.0045), fewer patients with known history of HF (58% vs 70%, *p* = 0.0248), arterial hypertension (55% vs 86%, *p* < 0.0001) and atrial fibrillation (44% vs 55%, *p* < 0.0001) and more patients with ischemic cause of HF (56% vs 47%, *p* = 0.0029). The population from the IRONMAN study were more frequently treated with sacubitril-valsartan (19% vs 7%, p < 0.0001) and betablockers (90% vs 84%, *p* = 0.0043), while mineralocorticoid-antagonists (54% vs 64%, *p* = 0.0007) and cardiac glycosides (11% vs 18%, *p* = 0.0011) were more frequently administered in AFFIRM-AHF. Cardiac resynchronization therapy was more frequently applied in IRONMAN (21% vs 5%, *p* < 0.0001) compared to AFFIRM-AHF.

**Table 2:** Comparison of baseline characteristics between usual care group and treatment group from IRONMAN and AFFIRM HF trials

Baseline characteristics	Usual care group			Treatment		
	IRONMAN (*n* = 568)	AFFIRM AHF (*n* = 550)	*P*- value	IRONMAN (*n* = 569)	AFFIRM AHF (*n* = 558)	*P*- value
Age, years	73.5 (67.1–79.1)	70.9 (11.1)	NA	73.2 (66.7–80.1)	71.2 (10.8)	NA
*Gender*						
Female	158 (28%)	250 (45%)	< 0.0001	142 (25%)	244 (44%)	< 0.0001
Male	410 (72%)	300 (55%)	< 0.0001	427 (75%)	314 (56%)	< 0.0001
BMI, kg/m^2^	28.3 (24.7–32.5)	28 (5.7)	NA	28.5 (24.7–32.6)	28.1 (5.6)	NA
*Race*						
White	524 (92%)	523 (95%)	0.05	519 (91%)	528 (95%)	0.02
Black	7 (1%)	/	NA	12 (2%)	/	NA
Asian	31 (5%)	22 (4%)	0.25	35 (6%)	26 (5%)	0.26
Other	6 (1%)	5 (1%)	0.80	3 (1%)	4 (1%)	0.68
Recruitment context	84 (15%)	550 (100%)	NA	80 (14%)	558 (100%)	NA
*Admitted to hospital* for HF and expected to survive to discharge						
*Admitted to hospital* for HF within past 6 months	102 (18%)	NA	NA	106 (19%)	NA	NA
*Outpatient* with raised natriuretic peptide concentration	382 (67%)	NA	NA	383 (67%)	NA	NA
*NYHA functional class*						
Class I	/	8 (1%)	NA	/	14 (3%)	NA
Class II	320 (56%)	240 (44%)	< 0.0001	328 (58%)	255 (46%)	< 0.0001
Class III	238 (42%)	277 (50%)	0.0045	230 (40%)	272 (49%)	0.0049
Class IV	10 (2%)	22 (4%)	0.0248	11 (2%)	16 (3%)	0.3052
Heart rate, beats per min	69 (40–79)	74.2 (12.8)	NA	70 (60–80)	74.5 (13.2)	NA
Systolic blood pressure, mm Hg	119 (106–132)	119.7 (15.6)	NA	119 (106–133)	119.8 (15.2)	NA
Left ventricular ejection fraction	35% (26–38)	32.7% (10)	NA	32% (25–37)	32.6% (9.6)	NA
*Medical history*						
Previous history of HF	324 (57%)	385 (70%)	< 0.0001	337 (59%)	405(73%)	< 0.0001
Hypertension	315 (55%)	471 (86%)	< 0.0001	297 (52%)	468 (84%)	< 0.0001
Diabetes	269 (47%)	243 (44%)	0.2864	252 (44%)	227 (41%)	0.2207
Atrial fibrillation	250 (44%)	305 (55%)	< 0.0001	284 (50%)	314 (56%)	0.0324
Ischemic cause of HF	316 (56%)	257 (47%)	0.0029	331 (58%)	265 (47%)	0.0003
*Device Therapy*						
Implantable cardioverter-defibrillator	72 (13%)	64 (12%)	0.5949	91 (16%)	67 (12%)	0.0540
Cardiac resynchronisation therapy	118 (21%)	30 (5%)	< 0.0001	125 (22%)	33 (6%)	< 0.0001
Haemoglobin, g/dl	12.1 (11.2–12.9)	12.1 (1.6)	NA	12.1 (11.2–12.8)	12.3 (1.6)	NA
Transferin saturation	15% (10–19)	14.2% (7.5)	NA	15% (11–20)	15.2% (8.3)	NA
Ferritin, μg/L	50 (30–85)	88.5 (68.6)	NA	49 (30–86)	83.9 (62.2)	NA

Data are mean (SD), *n* (%), or median (IQR), *NA *not applicable, *HF* heart failure, *NYHA* New York Heart Association, *BMI* body mass index

Population allocated to intervention (treatment with i.v. iron supplementation) from IRONMAN compared to the AFFIRM-AHF study included more men (75% vs 56%, *p* < 0.0001) and fewer women (25% vs 44%, *p* < 0.0001), more patients NYHA class II (58% vs 46%, *p* < 0.0001) and fewer with NYHA class III (40% vs 49%, *p* = 0.0049), fewer patients with known history of HF (59% vs 73%, *p* < 0.0001), arterial hypertension (52% vs 84%, *p* < 0.0001) and atrial fibrillation (50% vs 56%, *p* = 0.0324) and more patients with ischemic cause of HF (58% vs 47%, *p* = 0.0003). Population from intervention group from IRONMAN was more frequent treated with sacubitril-valsartan (23% vs 6%, *p* < 0.0001) and beta blockers (88% vs 81%, *p* = 0.0019), while loop diuretics (80% vs 88%, *p* < 0.0061) and mineralocorticoid receptor antagonists (57% vs 67%, *p* = 0.0004) were more frequently administered in population from AFFIRM-AHF study. Cardiac resynchronization therapy was more frequently applied in IRONMAN (22% vs 6%, *p* < 0.0001) compared to AFFIRM-AHF.

### Results from AFFIRM-AHF and IRONMAN trials

**Fig. 2:**
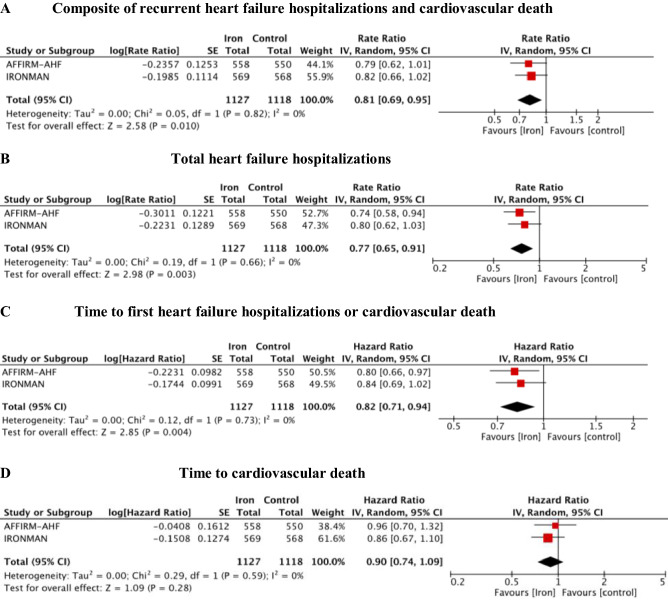
Forest plot for recurrent (**A**–**B**) and time-to-event (**C**–**D**) outcomes: (**A**) composite of recurrent heart failure hospitalizations and cardiovascular death; (**B**) total heart failure hospitalizations; (**C**) time to first heart failure hospitalizations or cardiovascular death; (**D**) time to cardiovascular death. IV, inverse variance; CI, confidence interval; SE, standard error

Treatment with i.v. iron supplementation compared to placebo reduced the composite endpoint of recurrent HF hospitalizations and CVD (RR = 0.81; 95% CI 0.69–0.95, *p* = 0.01, *I*^2^ = 0%) (Fig. 2A) and the risk of recurrent HF hospitalizations (RR = 0.77; 95% CI 0.65–0.91, *p* = 0.003, *I*^2^ = 0%) (Fig. 2B). NNT for the composite endpoint was 7 and for recurrent HF hospitalizations it was 8 over a weighted mean follow-up of 96 weeks. The meta-analysis had 73% power to detect a 19% RRR in the composite endpoint, while the summary result for the primary endpoint with FI of 94 and FQ of 0.041 was robust without heterogeneity (Table S2, Supplement).

**Fig. 3:**
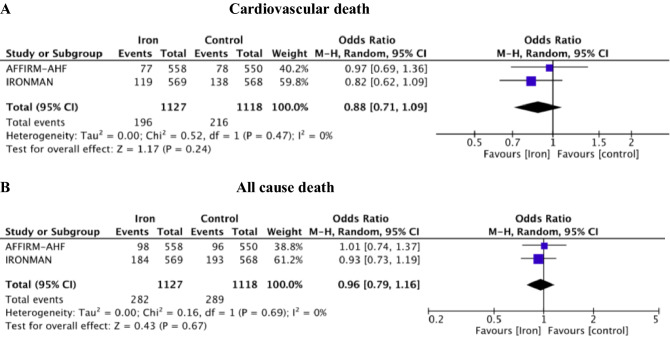
Forest plot for dichotomous outcome: **A** cardiovascular mortality; **B** all-cause mortality. *M–H* Mantel–Haenszel, *CI* confidence interval

FCM/FDI exhibit benefit on time to first HF hospitalization or CVD (HR = 0.82; 95% CI 0.71–0.94, *p* = 0.004, *I*^2^ = 0%) (Fig. 2C), but not on time to CVD alone (HR = 0.90; 95% CI 0.74–1.09, *p* = 0.28, *I*^2^ = 0%) (Fig. 2D) compared with usual care treatment. Treatment with i.v. iron numerically but not significantly reduced all-cause death (OR = 0.96; 95% CI 0.79–1.16, *p* = 0.67, *I*^2^ = 0%) (Fig. 3A) and CVD (OR = 0.88; 95% CI 0.71–1.09, *p* = 0.24, *I*^2^ = 0%) (Fig. 3B). The meta-analysis had 21% power to detect a 12% OR reduction in CVD, while summary result for endpoint CVD with RFI of 14 and RFQ of 0.006 was fragile without heterogeneity (Table S2, Supplement).

Subgroup analyses from AFFIRM-AHF[21] and IRONMAN [13] suggested that patients with ischemic heart disease might have more benefit from i.v. iron repletion on reduction of the primary endpoint (RR = 0.71; 95% CI 0.57–0.87, *p* = 0.001) compared to patients with non-ischemic heart disease (RR = 0.97; 95% CI 0.75–1.26, *p* = 0.83) as p for interaction of 0.06 showed a trend toward significance (Fig. S2, Supplement).

### COVID-19 prespecified analysis from IRONMAN and AFFIRM-AHF trial

**Fig. 4:**
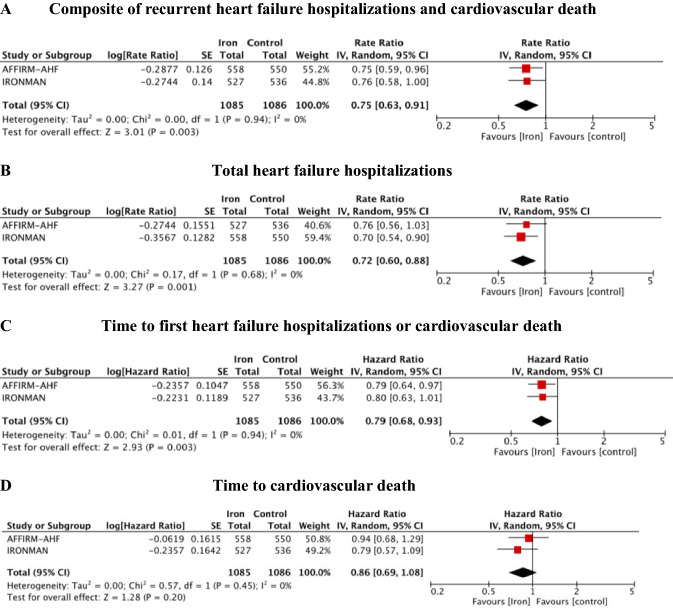
Forest plot for recurrent (**A**–**B**) and time-to-event (**C**–**D**) outcomes determined by pooling the data from COVID prespecified analysis: **A** composite of recurrent heart failure hospitalizations and cardiovascular death; **B** total heart failure hospitalizations; **C** time to first heart failure hospitalizations or cardiovascular death; **D** time to cardiovascular death. *IV* inverse variance, *CI* confidence interval, *SE* standard error

Treatment with FCM/FDI compared to placebo reduced the composite endpoint of recurrent HF hospitalizations and CVD (RR = 0.75; 95% CI 0.63–0.91, *p* = 0.003) (Fig. 4-A) and the risk of recurrent HF hospitalizations (RR = 0.72; 95% CI 0.60–0.88, *p* = 0.001) (Fig. 4-B). There was no heterogeneity (*I*^2^ = 0%) for any outcome between the trials. NNT for the composite outcome was 11 over a weighted mean of follow-up of 96 weeks. The meta-analysis had 85% power to detect a 25% RRR in the composite endpoint, while summary result for primary endpoint with FI of 98 and FQ of 0.045 was robust (Table S2, Supplement).

Iron IV showed benefit on time to first HF hospitalization or CVD (HR = 0.79; 95% CI 0.68–0.93, *p* = 0.003) (Fig. 4-C) but not on time to CVD alone (HR = 0.86; 95% CI 0.69–1.08, *p* = 0.20) (Fig. 4D). There was no heterogeneity (*I*^2^ = 0%) for any outcome between the trials. The meta-analysis had 24% to detect a 14% HRR of time to CVD. Summary result for endpoint CVD with RFI of 10 and RFQ of 0.004 was fragile (Table S2, Supplement).

### Data from all eligible trials

Analysis of all eligible trials confirmed findings from the main analysis. Treatment with FCM/FDI compared to placebo reduced the composite endpoint of recurrent HF hospitalizations and CVD (RR = 0.77; 95% CI 0.66–0.90, *p* = 0.0008) (Fig. S3-A, Supplement) and the risk of recurrent HF hospitalizations (RR = 0.72; 95% CI 0.57–0.92, *p* = 0.007) (Fig. S3-B, Supplement). There was no relevant heterogeneity for any outcome neither for composite outcome (*I*^2^ = 0%) nor for recurrent HF hospitalizations (*I*^2^ = 39%). NNT for composite outcome was 6 and for recurrent HF hospitalizations was 7 over a weighted mean follow-up of 47 weeks. The meta-analysis had power of 91% to detect 23% RRR in composite outcome. Summary result for primary endpoint with FI of 147 and FQ of 0.045 was robust (Table S2, Supplement).

Treatment with FCM/FDI showed significant benefit on time to first HF hospitalization or CVD (HR = 0.79; 95% CI 0.67–0.92, *p* = 0.003) (Figure S3-C, Supplement) but not on time to CVD (HR = 0.89; 95% CI 0.74–1.08, *p* = 0.24) (Figure S3-D, Supplement). There was no relevant heterogeneity for any outcome neither for time to first HF hospitalization or CVD (*I*^2^ = 25%) nor for time to CVD (*I*^2^ = 0%). Effects of iron supplementation were neutral concerning all-cause mortality (OR = 0.94; 95% CI 0.78–1.13, *p* = 0.49) (Figure S4-A, Supplement) and CVD (OR = 0.87; 95% CI 0.71–1.07, *p* = 0.18) (Figure S4-B, Supplement). There was no heterogeneity (*I*^2^ = 0%) for any outcome between the trials. The meta-analysis had power of 10% to detect 14% ORR in CVD, while summary result of meta-analysis for CVD with RFI of 7 and RFQ of 0.002 was fragile (Table S2, Supplement).

### Safety analysis

Data regarding safety endpoints were explored according to the available data from four trials (IRONMAN, AFFIRM-AHF, FAIR-HF and CONFIRM-HF). There was no relevant difference between iron supplementation and placebo for infections (OR = 0.85; 95% CI 0.71–1.02, *p* = 0.09) general or injection-site related disorder (OR = 1.39; 95% CI 0.88–2.19, *p* = 0.16, *I*^2^ = 30%) and vascular disorder (OR = 0.84; 95% CI 0.57–1.25, *p* = 0.39) (Figure S5 A–C, Supplement). There was no relevant heterogeneity for any safety outcome neither for infections (*I*^2^ = 0%), nor for general or injection-site related disorder (*I*^2^ = 30%), nor for vascular disorder (*I*^2^ = 0%).

## Discussion

The key findings of our analysis are the following: (i) IV iron repletion with FCM or FDI compared to placebo reduced the composite endpoint of recurrent HF hospitalization and CVD in HF patients with ID; (ii) effects of FCM or FDI concerning all-cause death or CVD are based on current level of data indeterminate; (iii) use of FCM and/or FDI was safe. In addition, findings concerning the composite endpoint were very robust while findings concerning CVD were fragile. There was no relevant heterogeneity for any outcome between the trials with FCM and those with FDI.

One reason to perform meta-analyses is to increase the power of trials with borderline patient and limited event numbers [22]. It has been recognized that the COVID-19 pandemic had unpredictable and adverse impacts on conduction, results, clinical outcome as recruitment of patients and adequate follow-up [14]. To overcome these obstacles in AFFIRM-AHF and IRONMAN prespecified COVID-19 sensitivity analyses were planned which censored patients at the date when the first COVID-19 patient was reported (AFFIRM-AHF) [12] or at the start of the national lockdown in the United Kingdom (IRONMAN)[13]. COVID-19 pre-specified analyses showed significant reductions in the primary endpoint with FCM/FDI compared to placebo.

Meta-analysis of the data from AFFIRM-AHF [12] and IRONMAN [13] showed that treatment with FCM/FDI reduced the composite endpoint of recurrent HF hospitalizations and CVD, which was mainly driven by reduction of recurrent HF hospitalizations. This finding was confirmed in both sensitivity analyses by meta-analysis of the data from prespecified COVID-19 analyses and from the all eligible studies investigating iron repletion with FCM/FDI in HF patients. There was no sign of statistical heterogeneity across the included trials, as *I*^2^ for composite endpoint was zero in main and both sensitivity analyses. Main analysis had enough power (73%) to detect observed RRR concerning primary endpoint as well as COVID-19 analysis (85%) and analysis of all eligible trials (91%). Summary findings for primary endpoint in main analysis were very robust with FI of 94, indicating that 94 events of primary endpoint should be added to FCM/FDI group or subtracted from placebo group to render the result negative for this outcome, and FQ of 0.041, indicating 41 events per 1000 patients added to FCM/FDI group needed to render the results negative for this outcome. Robustness of observed results from all eligible trials was with FI of 147 and FQ of 0.045 even more pronounced. NNT for composite endpoint was 7 in main analysis and 6 in analysis with all eligible trials, indicating that 6–7 patients should be treated to avoid one composite outcome, thereby showing a remarkable efficacy profile.

HF is one of the leading causes for hospitalization being responsible for about 5% emergency hospital admissions worldwide [23]. Median number of HF discharges per million people in Europe amounts 2671 (IQR 1771–4317) with median length of hospital stay of 8.50 days (IQR 7.38–10) [24]. According to the same source median prevalence of HF approximate 17.2 per 1000 people [24]. Taking these figures into account it becomes obvious to what extent HF poses a burden on health-care givers and national economies worldwide. Therefore, reduction in HF rehospitalizations is of paramount importance as one of the mechanisms in improvement of outcome in HF patients.

Our analysis suggests a numerical but no significant effect of i.v. iron repletion on CVD or all-cause death in HF patients. Incident rates of CVD were numerically lower by 2% in patients receiving FCM/FDI compared with usual care group. Of note, both trials were not powered concerning these outcomes. In line, calculated power of meta-analyses for CVD was too low across all explored populations, thus not allowing us to make any reliable conclusion regarding effects of FCM/FDI on this outcome. However, summary findings of meta-analyses for endpoint CVD were fragile (RFI of 14, i.e., 14 events of CVD added to placebo or subtracted from FCM/FDI group of patients needed to render the result positive for CVD, and RFQ of 6, i.e., indicating 6 events per 1000 patients added to the placebo group needed to render the results positive for CVD). Fragility of the meta-analysis summary results for CVD was even more pronounced in analysis where all eligible trials were explored (RFI of 7 and RFQ of 0.002). Furthermore, duration of the follow-up could have been too short in AFFIRM-AHF. Notably, incidence rate reduction was more expressed with iron repletion compared to placebo during longer follow-up as presented in IRONMAN trial. However, this speculate should be regarded as hypothesis generating. Summarized, according to the totality of current available evidence effects of FCM/FDI on CVD are debatable and indeterminate.

Iron represents the essential element for transport and storage of oxygen, especially in cells with high energy demand like skeletal and heart muscle cells [5]. Iron deficiency independently of anemia negatively affects oxidative metabolism, cellular energetic [25] and immune mechanisms which result in decreased oxygen storage in myoglobin and reduced myocardial oxygen capacity leading to mitochondrial and left ventricle dysfunction [5]. Reduced myocardial iron correlates with reduced reactive oxygen species (ROS) protecting enzymes and mitochondrial oxygen consumption [5, 25]. These pathological mechanisms contribute to myocardial dysfunction and adverse remodeling which further deteriorate functional capacity of HF patients and promote worsening of HF. Positive findings of pre-specified COVID-19 analyses of AFFIRM-AHF and IRONMAN on outcome of HF patients with ID, strengthen by results of this analysis unequivocally point out that iron substitution with FCM/FDI should be recognized as a life-saving therapy for appropriate patients (those with ID).

Populations enrolled in the AFFIRM-AHF and IRONMAN differed slightly concerning gender, load of comorbidities, HF symptoms (NYHA class) and background medication. AFFIRM-AHF trial enrolled patients being hospitalized for acute HF while IRONMAN included predominantly (67%) ambulatory HF patients. This makes its comparisons difficult. Nevertheless, composite outcome was similar without statistical heterogeneity (*p* for Cochran Q = 0.82; *I*^2^ = 0%). Therefore, benefit from i.v. iron repletion remains consistent despite some different presentations of HF patients.

Both substances explored in this analysis (FCM and FDI) are comparable regarding their ability to restore iron stores based on increases in ferritin and transferrin saturation [12, 13]. However, there are some discussions about different safety profiles between FCM and FDI related to hypophosphatemia. It has been shown that FCM is associated with higher incidence of hypophosphatemia compared to FDI in patients (mainly women > 90%) with ID anaemia that lasted up to 35 days [26] as in patients with inflammatory bowel disease and ID anaemia [27]. Of note, patients with HF were underrepresented in these randomized trials. In a small (*n* = 23) single-center study FCM was investigated in HFrEF patients with (CKD +) and without chronic kidney disease (CKD-) [28]. Interestingly, significant serum phosphate decreases were present only in CKD(-) patients, while in both groups transient hypophosphatemia (< 0.8 mmol/l) was observed (9/11 in CKD(-) and 5/12 in CKD( +) patients) in parallel with a decrease in the levels of 1,25-OH vitamin D. Hypophosphatemia following i.v. iron replacement has been induced by increased secretion of fibroblast growth factor (FGF) 23 that leads to increased urinary phosphate excretion and decreased concentration of active vitamin D [28, 29]. Dose, repetitive iron infusions, severity of ID, increasing age and vitamin D deficiency among others have been identified as predisposing factors for development of this side-effect [30]. Nevertheless, it remains unclear whether and at what extent this might have negative long-term effects on HF patients. In the AFFIRM-AHF study from week 12 to week 52 level of change regarding serum phosphate was similar (literary one patient in each group experienced hypophosphatemia) between the FCM and placebo group [12] while in IRONMAN study data regarding serum phosphate were not collected [13]. A placebo controlled study is needed to further explore potential hazard.

Administration of FCM and FDI was well tolerated. There was no difference between treatment and control group in terms of infection, general or injection-site disorder or vascular disorder.

## Limitations

There are several limitations that need to be acknowledged. This is a post-hoc meta-analysis using the data provided in the official publications and not the individual patient-level data, which does not allow identifying possible covariates that might have impact the final results. Dosing regimen concerning initial dose, maintenance doses, probably total doses as well timing of control of iron values and iron re-administration varied substantially between the trials depending mostly on the duration of the trials which all might have affected results.

## Conclusion

In summary, the totality of evidence of ID treatment trials suggests that the use of FCM/FDI in patients with HF and ID is safe and associated with reduced rate of composite of recurrent hospitalizations for heart failure and CVD. The data are robust derived from analyses with sufficient power to detect observed treatment effects and do not show relevant heterogeneity between the trials with the two iron derivates.
